# Toxigenic profiles and trinucleotide repeat diversity of *Fusarium* species isolated from banana fruits

**DOI:** 10.1080/13102818.2014.995519

**Published:** 2015-01-08

**Authors:** Mousa Abdullah Alghuthaymi, Ali Hassan Bahkali

**Affiliations:** ^a^ Biology Department, Science and Humanities College, Shaqra University, Shaqra, Saudi Arabia; ^b^ Botany and Microbiology Department, Faculty of Science, King Saud University, Riyadh, Saudi Arabia

**Keywords:** banana fruits, *Fusarium*, microsatellite markers, ISSR analysis, mycotoxin

## Abstract

Infesting *Fusarium* species isolated from banana fruit samples were identified and quantified by morphological, mycotoxicological and molecular tools. A total of 19 *Fusarium* isolates were obtained: *F. semitectum* was most predominant (26%), followed by *F. proliferatum* (16%), *F. circinatum* (16%), *F. chlamydosporum* (10.5%), *F. solani* (10.5%), *F. oxysporum* (10.5%) and *F. thapsinum* (5%). Fumonisin B1, deoxynivalenol and zearalenone contents were assayed by high-performance liquid chromatography (HPLC). Seventeen isolates, belonging to *F. chlamydosporum*, *F. circinatum*, *F. semitectum*, *F. solani*, *F. thapsinum*, *F. proliferatum* and *Fusarium* spp., produced mycotoxins when cultured on rice medium. Fumonisin was produced by all of the studied *Fusarium* isolates, except *F. oxysporum*, at a concentration of over 1 μg**/**mL. *F. citrinium* isolates 4 and 5 and *F. solani* isolate 3 were the most potent producers of deoxynivalenol. We compared the 19 *Fusarium* isolates based on the bands amplified by 10 microsatellite primers. Of these, seven primers, (TCC)5, (TGG)5, (GTA)5, (ATG)5, (TAC)5, (TGC)5 and (TGT)5, yielded a high number of bands and different mean number of alleles. The similarity level between isolates was calculated using a simple matching coefficient. Dendrograms were constructed by the unweighted pair-group method with arithmetical averages (UPGMA). Two main clusters were observed. The interspecific genetic similarity between *Fusarium* spp. isolates was between 40% and 58% and the intraspecific similarity from 58% to 100%, indicating a high degree of genetic diversity in the tested isolates. Some unexpected genetic similarities were observed among the isolates, indicating non-agreement between morphological and molecular identification of the isolates.

## Abbreviations

AT: Annealing temperature
DON: Deoxynivalenol
FB1: Fumonisin B1
HPLC: High-performance liquid chromatography
ISSR: Inter-simple sequence repeats
MP: Microsatellite primers
MP-PCR: Microsatellite-primed PCR
MW: Molecular weight
PCR: Polymerase chain reaction
SNA: Synthetic nutrient agar
UPGMA: Unweighted pair-group method with arithmetical averages
ZEA: Zearalenone

## Introduction

The edible banana (*Musa* spp.) is a fruit plant produced in several tropical countries throughout the world and in many of these countries its cultivation and marketing play a vital economic and social role.[1] Some fungal species colonize the banana fruits and produce mycotoxins.[2] To date, there have been only a few published reports on secondary metabolites produced by *Fusaria* in infested banana fruits.[3–7] Some *Fusarium* isolates are mycotoxin producers and, in some cases, an isolate can produce various toxic metabolites.[7] Thus, detection tools for multi-mycotoxin analysis that allow the evaluation of the toxigenic potential of *Fusarium* spp. growing in and on bananas would be very valuable.

The most important mycotoxin-producing *Fusarium* species, *F. verticillioides* (*Gibberella moniliforme*, *G. fujikuroi* mating population A), has been isolated from bananas in distant locations, such as India,[8] the Windward Islands,[9] Panama, Ecuador and the Canary Islands.[10] The most toxicologically important *Fusarium* mycotoxins in terms of plant health and productivity are deoxynivalenol (DON), zearalenone (ZEA), fumonisins (fumonisin B1, FB1) and moniliformin.[4] The morphological identification of *Fusarium* spp., which are typically observed in pure culture, is quite laborious and time consuming. This method requires the skills of a trained plant pathologist and it is not always suitable for fungal species identification.[11] Information about the genetic diversity and population structure of pathogen *Fusarium* species is crucial for designing an appropriate plant disease management. The need for alternative and/or complementary taxonomic techniques for the rapid and accurate identification of *Fusarium* species is, therefore, high, especially in the tropical countries where the species are abundant.

The main objectives of the present study were: (1) to measure the occurrence and levels of FB1, DON and ZEA in *Fusarium* isolates collected from banana fruits imported into Saudi Arabia in 2012 and (2) to provide a molecular characterization of these isolates, using trinucleotide repeat analysis.

## Materials and methods

### *Fusarium* isolation and purification

The plant material consisted of commercially available banana fruits originating from Costa Rica, Ecuador, Brazil and Malaysia, imported into Saudi Arabia. The banana fruits were examined for the presence of *Fusarium* species in 2012. Ten samples (0.5 cm × 0.5 cm pieces) of the banana fruits that showed rot symptoms were disinfected by dipping into a 4% sodium hypochlorite solution for 4 min, followed by rinsing with sterile distilled water three times for 1 min. Extra water in the examined samples was removed with the use of a sterile filter paper. Potato dextrose agar (PDA) was used for fungal isolations. The inoculated Petri dishes were incubated at 25 °C for 7 days. All samples were inoculated in triplicate. For most of the isolates, single-spore cultures were obtained according to Leslie and Summerell.[12] Identification was done by culturing the isolates on synthetic nutrient agar (SNA; 1.0 g/L of KH_2_PO4, 1 g/L of KNO_3_, 0.5 g/L of MgSO_4_.7H_2_O, 0.5 g/L of KCl, 0.2 g/L of glucose, 0.2 g/L of sucrose and 20.0 g/L agar; prepared in distilled water) and observation of cultural and microscopic characteristics.[13,14] Fungal cultures were examined microscopically under low magnification (×100 to ×200) to study the morphological structures of the aerial mycelia. *Fusarium* cultures were maintained on PDA medium at 4 °C and then stored as spore suspensions in 15% glycerol at −80 °C.

### Fumonisin B1 (FB1), deoxynivalenol (DON) and zearalenone (ZEA) quantification

For mycotoxin production assays, the 19 *Fusarium* isolates (Table 1) were cultured in 250 mL Erlenmeyer flasks containing 50 mL of rice culture medium (50 g of rice flour per 500 mL deionized water). The top of each flask was covered with aluminum foil, autoclaved and was allowed to cool down in a hood for about 40 min. Mycelia from PDA plates were gently scrapped from the agar, using sterile scalpels. Then, each flask was inoculated by adding the mycelia from the *Fusarium* isolates. The flasks were incubated in a regulated room at 24 °C for about 28 days. Mycelia were harvested by filtration through Whatman no. 1 paper. The dry mycelium was ground to fine powder using a coffee blender with ethanol cleaning between samples. The ground samples were stored at 0 °C until use.

**Table 1.** Mycotoxins produced by 19 *Fusarium* isolates which were isolated from post-harvest diseased banana fruits from the Saudi Arabia market (2012).

		Fusarium mycotoxins					
Isolate code	Species	FB1 (μg/mL)	Limit^‡^ for FB1 (%)	ZEA (μg/mL)	Limit for ZEA (%)	DON (μg/mL)	Limit for DON (%)
1.	*F. chlamydosporum*	1.012	+1.20	0.100	−50.00	0.312	−58.40
2.	*F. chlamydosporum*	1.322	+32.20	0.230	+15.00	0.521	−30.53
3.	*F. circinatum*	1.065	+6.50	0.150	−25.00	0.319	−57.47
4.	*F. circinatum*	1.852	+85.20	0.855	+325.00	0.829	+10.53
5.	*F. circinatum*	1.801	+80.10	0.890	+345.00	0.811	+8.13
6.	*F. oxysporum*	nd	nd	nd	nd	nd	nd
7.	*F. oxysporum*	nd	nd	nd	nd	nd	nd
8.	*F. semitectum*	1.414	+41.40	0.223	+11.50	0.549	−26.80
9.	*F. semitectum*	1.126	+12.60	0.090	−55.00	0.298	−60.27
10.	*F. semitectum*	1.349	+34.90	0.215	+7.50	0.578	−22.93
11.	*F. semitectum*	1.789	+78.90	0.919	+359.50	0.815	+8.67
12.	*F. semitectum*	1.657	+65.70	0.764	+282.00	0.678	−9.60
13.	*F. solani*	1.561	+56.10	0.771	+285.50	0.664	−11.47
14.	*F. solani*	1.823	+82.30	0.893	+346.50	0.801	+6.80
15.	*F. thapsinum*	1.801	+80.10	0.890	+345.00	0.811	+8.13
16.	*F .proliferatum*	1.414	+41.40	0.223	+11.50	0.549	−26.80
17.	*F. proliferatum*	1.834	+83.40	0.900	+350.00	0.823	+9.73
18.	*F. proliferatum*	1.779	+77.90	0.912	+356.00	0.809	+7.87
19.	*Fusarium* spp.*	1.802	+85.20	0.850	+325.00	0.820	+10.53

Note: nd = not detected.

*This isolate was considered unknown. It did not have the ability to form any spores.

^‡^Mycotoxins were determined by using HPLC and compared with international regulatory limits for *Fusarium* mycotoxins.

### High-performance liquid chromatography

High-performance liquid chromatography (HPLC) was used to detect the following mycotoxins: FB1, DON and ZEA. Standards of these toxins were purchased from Sigma Chemical Company (St. Louis, MO, USA) and stored at 4 °C in darkness. The procedure was performed as previously described in detail in [15]. The detection limits for the tested mycotoxins are shown in Table 1.

### Fungal growth and DNA isolation

DNA was purified from the mycelia of the 19 isolates. The mycelia were grown in 5 mL potato dextrose broth (PDB; Gibco, USA) by shaking the culture at 130 r/min at 24 °C for 6 days. Total genomic DNA was purified from fresh mats of *Fusarium* isolates. Total DNA was extracted by a modified mini-prep standard method.[16]

Fungal mycelium (100 mg) was homogenized in 400 μL of sterile salt homogenizing buffer (200 mmol/L Tris–HCl, pH 8.5, 250 mmol/L NaCl, 25 mmol/L ethylenediaminetetraacetic acid (EDTA), 0.5% sodium dodecyl sulphate (SDS)). Then, 6 μL of 20 mg/mL RNase A (20 mg/mL final concentration) were added and mixed well. The samples were incubated at 65 °C for 10 min, after which 130 μL of 3 mol/L sodium acetate (pH 5.2) was added to each sample. Then the samples were vortexed for 30 s at maximum speed, and incubated at −20 °C for 10 min. The lysate was centrifuged at 13,000 r/min at 4 °C for 15 min. The supernatant was transferred to fresh tubes. An equal volume of isopropanol was added to each sample, mixed well, and the samples were incubated at −20 °C for 10 min. The samples were then centrifuged for 20 min at 4 °C and 6000 r/min. The DNA pellets were washed twice with 700 μL of washing solution (100% and 70% ethanol, respectively). The DNA pellets were subsequently air-dried in an oven at 40 °C for at least 10 min. The resultant DNA pellet was resuspended in 100 μL of 1× Tris–EDTA (TE) buffer (10 mmol/L Tris–HCl, 1 mmol/L EDTA, pH 8.0).

### Microsatellite-primed polymerase chain reaction (MP-PCR)

The PCR mixture contained 15 pmol/L primer, 10 mmol/L Tris–HCl (pH 9.0), 50 mmol/L KCl, 0.1% (v/v) Triton® X-100, 2.5 mmol/L MgCl_2_, 200 μmol/L of each deoxynucleoside triphosphate (dNTP), 1 U of *Taq* polymerase (JenaBioscience, Germany) and 5 ng of template DNA in a total volume of 25 μL. The PCR reactions were run in a Thermocycler (ESCO, Korea) as follows: 94 °C for 2 min, followed by 40 cycles of 94 °C for 1 min, 45–55 °C (depending on primers used) for 90 s and 72 °C for 2 min, with a final extension at 72 °C for 6 min. PCR amplification with each primer was repeated at least twice to check the reproducibility of DNA profiles. Trinucleotide microsatellite primers were used in the present study and their length, GC content, molecular weight, motifs, optimum annealing temperature and size range of fragments are shown in Table 2. Amplification products were separated by 1.5% (w/v) agarose gel electrophoresis and stained with ethidium bromide. An unweighted pair-group method with arithmetical averages (UPGMA) dendrogram was constructed by using UVI-BandMap software analysis (UVTEC, UK).

**Table 2.** Tri-microsatellite primers used in the present study.[17]

	Primers	Length (bp)	GC content (%)	MW	Optimum AT (°C)	Range of fragment size (bp)
Nucleotides repeats	(AGG)5	15	66.7	4796.1	52	350–2000
	(TCC)5	15	66.7	4350.8	50	400–2000
	(ACG)5	15	66.7	4596.0	50	400–2000
	(TGG)5	15	66.7	4751.1	50	350–1500
	(GTA)5	15	33.3	4671.1	40	600–2000
	(ATG)5	15	33.3	4671.1	50	200–3000
	(TAC)5	15	33.3	4470.9	40	700–1500
	(TGC)5	15	66.7	4550.9	40	1000–1800
	(GCT)5	15	66.7	4550.9	52	600–2000
	(TGT)5	15	33.3	4626.0	40	400–1800

Note: MW = molecular weight; AT = annealing temperature.

## Results and discussion

### *Fusarium* species

**Figure 1.**
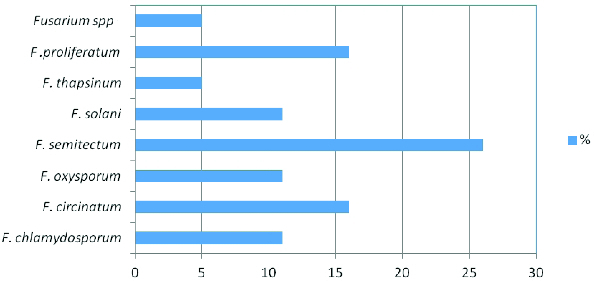
Percentage of *Fusarium* spp. in post-harvest diseases of banana fruits.

*Fusarium* rot from banana fruits collected from four different countries was screened in 2012. The results from the different isolation experiments indicated that there were 19 fungal isolates belonging to the *Fusarium* genus. The following *Fusarium* species were isolated: *F. semitectum* (five isolates), *F. proliferatum* (three isolates), *F. circinatum* (three isolates), *F. chlamydosporum* (two isolates), *F. solani* (two isolates), *F. oxysporum* (two isolates) and *F. thapsinum* (one isolate) (Figure 1). In this respect, *F. semitectium*, as the predominant cause of *Fusarium* rot in stored banana fruits, is a distinctive wound pathogen. Twenty samples were collected from each location; four replicates were collected for every 20 samples. Other studies show that *F. semitectum* is commonly isolated from soil [17] and from diverse aerial plant parts in tropical and subtropical areas, e.g., from banana fruits.[18,19] In previous studies, based on morphological and molecular characterization, seven strains of *Fusarium* isolated from rotten banana fruits and imported into Japan from Mexico were identified as *F. verticillioides.*[6,20,21] *F. proliferatum* has also been isolated from banana samples from 12 localities in Sri Lanka.[22]

### Chemical analyses of toxin-producing isolates

In the present study, toxigenic *Fusarium* isolates were more prevalent than non-toxigenic isolates. Only two *F. oxysporum* isolates were found to be non-toxigenic. *F. citrinium* isolates 4 and 5 as well as *F. solani* isolate 14, *Fusarium* spp. isolate 19 and *F. proliferatum* isolate 17 were some of the highest producers of FB1 toxin, while the other isolates had intermediate toxicity (Table 1). For DON toxin the same trend was observed as that for FB1 for most isolates were the detected FB1 exceeded the 0.75 μg/mL limit defined in the international regulations. *F. citrinium* isolates 4 and 5, *F. semitectum* isolate 11, as well as *Fusarium* species and *F. proliferatum* isolates 17 and 18, were among the most potent producers of ZEA toxin. There are reports that fumonisin toxin can be produced by *Fusarium* isolates in a concentration exceeding 1 μg/mL.[23–25] In addition, *F. moniliforme* isolates from banana fruit in India produced trichothecenes and ZEA.[3,26] DON, ZEA, T-2 toxin and fumonisin are all produced by representatives of the *Fusarium* genus. Crops in tropical and subtropical countries are more susceptible to infestation than those in temperate climate regions, since the high humidity and temperature in tropical and subtropical areas provide optimal conditions for toxin formation.[27–29] The toxigenic potential of many *Fusarium* species emphasizes the need for accurate identification on the species level.[29] *Fusarium* species can be identified based on morphology alone.[12] However, identification based on molecular data is considered more reliable and accurate than morphological identification and has become much more important in diagnostics of the fungi from the genus *Fusarium*.[30]

### Trinucleotide repeat diversity

We compared the amplified bands and mean number of alleles amplified from 19 *Fusarium* species with 10 microsatellite primers. Of the 10 primers tested, seven primers, (TCC)5, (TGG)5, (GTA)5, (ATG)5, (TAC)5, (TGC)5 and (TGT)5, yielded a high number of bands and different mean number of alleles in the 19 *Fusarium* spp. isolates studied by us. The other three primers, (AGG)5, (ACG)5 and (GCT)5, amplified a small number of total bands and mean number of alleles in the studied *Fusarium* spp. isolates (Table 3). In a previous study, these 10 primers gave clear multiple banding patterns and also showed high polymorphism among different fungal species.[17]

**Table 3.** Total number of bands and mean number of alleles amplified from 19 *Fusarium* species isolates with 10 microsatellite primers (MP).

	Number of alleles amplified from different *Fusarium* species																				Mean number
Primers	*F.ch*1	*F.ch*1	*F.ci*1	*F.ci*2	*F.ci*3	*F.ox*1	*F.ox*2	*F.se*1	*F.se*2	*F.se*3	*F.se*4	*F.se*5	*F.so*1	*F.so*2	*F.th*	*F.pr*1	*F.pr*2	*F.pr*3	*F.sp*	TB	of alleles
(AGG)5	3	5	2	6	4	2	3	5	7	2	4	6	3	5	1	4	5	3	6	76	4
(TCC)5	7	5	7	6	10	3	7	9	5	5	3	7	4	8	6	2	4	4	3	105	5.5
(ACG)5	3	4	4	4	4	3	7	3	4	3	5	2	4	3	5	4	3	3	4	72	3.8
(TGG)5	4	8	5	7	4	6	10	5	7	3	6	11	6	9	7	4	8	6	9	125	6.6
(GTA)5	6	9	7	7	8	12	11	6	8	12	6	13	7	9	7	12	10	9	11	170	8.9
(ATG)5	5	4	6	5	4	7	7	6	8	4	4	3	8	5	3	9	8	2	3	101	5.3
(TAC)5	7	11	9	7	12	10	11	11	8	6	8	12	12	9	6	12	11	7	9	178	9.3
(TGC)5	6	6	9	8	6	5	7	4	7	8	6	9	4	3	3	5	5	4	7	112	5.9
(GCT)5	2	2	5	5	5	3	3	3	2	2	3	4	2	5	4	4	2	3	2	61	3.2
(TGT)5	11	9	14	11	13	7	14	8	9	10	11	13	9	15	7	13	10	10	7	201	10.6

Note: TB = total number of bands, *F.ch* = *F. chlamydosporum*, *F.ci* = *F. circinatum*, *F.ox* = *F. oxysporum*, *F.se* = *F. semitectum*, *F.so* = *F. solani*, *F.th* = *F. thapsinum*, *F.pr* = *F. proliferatum*, *F.sp* = *Fusarium* species.

**Figure 2.**
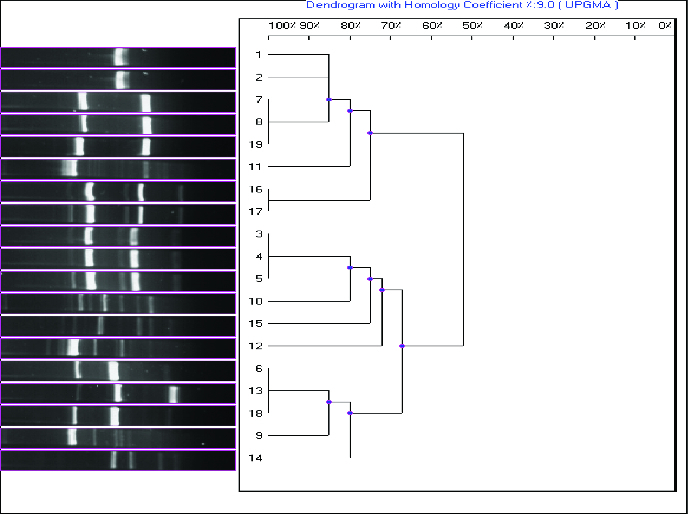
Dendrogram of the 19 *Fusarium* isolates constructed after cluster analysis of the digitized (GCT)5-MP-PCR fingerprints with UPGMA. Note: Lanes from 1 to 19 are: *F. chlamydosporum*, *F. chlamydosporum*, *F. circinatum*, *F. circinatum*, *F. circinatum*, *F. oxysporum*, *F. oxysporum*, *F. semitectum*, *F. semitectum*, *F. semitectum*, *F. semitectum*, *F. semitectum*, *F. solani*, *F. solani*, *F. thapsinum*, *F .proliferatum*, *F. proliferatum*, *F. proliferatum* and *Fusarium* spp.

It is noteworthy that using this technique, the isolates from 8 to 15 yielded isolate-specific patterns. UPGMA analysis of the (GCT)5 data separated the *Fusarium* spp. isolates into two main groups, each of which shared about 20% similarity. Among the studied *Fusarium* spp. isolates, the interspecific similarity ranged from 50% to 65% and the intraspecific comparisons showed from 65% to 100% similarity. The three *F. circinatum* isolates (3, 4 and 5) shared a high genetic similarity value (100%). Two of the *F. proliferatum* isolates (16 and 17) also shared a high genetic similarity value (100%), but the third *F. proliferatum* isolate (isolate 18) was clustered separately from them (Figure 2). This suggests that, in some cases, there is no clear-cut relation between molecular and morphological identification for *Fusarium* species.

Microsatellites have been used as molecular markers in numerous PCR fingerprinting studies for species genotyping of a variety of filamentous fungi, without prior knowledge of the abundance and distribution of these microsatellites in the investigated fungal genomes.[26,31–33] Microsatellites have found application in variability analyses in *Fusaria*, for instance, *F. oxysporum* f. sp. *ciceris* races [32,34] and *Fusarium oxysporum* f. sp. *radicis-lycopersici*.[33,34]

In the present study, some microsatellite-primed PCR (MP-PCR) markers revealed a high level of polymorphism among different isolates of *Fusarium* spp.[34] The dendrogram of inter-simple sequence repeats (ISSR) analysis grouped the 19 isolates into two major clusters. Ten primers were tested and each primer enabled a clear differentiation of all the tested species. However, the tested primers did not distinguish between mycotoxin producers and non-producers. Thus, a wide survey is needed for additional testing with PCR tools for discrimination of the toxigenic *Fusarium* isolates contaminating banana fruits in Saudi Arabia. Hence, there is a need to develop a simple and fast method for the identificaion of toxigenic fungi, and especially for distinguishing between producers and non-producers of toxins. Mapping of toxins on a phylogenetic tree can be very difficult due to misidentification of species and the complexity involved in toxin analyses.[35] Some unexpected genetic similarities that were observed among the isolates indicated non-agreement between morphological and molecular identification of the isolates.

## Conclusions

The predominant *Fusarium* species isolated from banana fruits were *F. semitectum*, followed by *F. proliferatum*, *F. circinatum* and *F. chlamydosporum*. Eighty-nine percent of the *Fusarium* isolates wеre capable of mycotoxin production. Trinucleotide repeat primers did not distinguish between mycotoxin producers and non-producers. Thus, an extensive survey is needed for further confirmation of this PCR method in discriminating toxigenic *Fusarium* isolates. To the best of our knowledge, this is the first report on mycotoxin production by *Fusarium* species that contaminate banana fruits imported into Saudi Arabia from different banana-growing areas.

## Disclosure statement

No potential conflict of interest was reported by the authors.
